# Exploring the Predictive Role of 11‐Oxyandrogens in Diagnosing Polycystic Ovary Syndrome

**DOI:** 10.1002/edm2.70022

**Published:** 2025-01-15

**Authors:** Armaiti Parvez Mody, Maya Beth Lodish, Richard Joseph Auchus, Adina F. Turcu, Fei Jiang, Heather Gibson Huddleston

**Affiliations:** ^1^ Division of Pediatric Endocrinology University of California Davis California USA; ^2^ Division of Pediatric Endocrinology University of California San Francisco California USA; ^3^ Division of Metabolism, Endocrinology and Diabetes University of Michigan Ann Arbor Michigan USA; ^4^ Division of Epidemiology & Biostatistics University of California San Francisco California USA; ^5^ Division of Reproductive Endocrinology and Infertility University of California San Francisco California USA

**Keywords:** 11‐ketotestosterone, 11‐oxygenated C19 steroids, androgens, hyperandrogenism, polycystic ovary syndrome

## Abstract

**Context:**

Hyperandrogenism is a hallmark of polycystic ovary syndrome (PCOS), yet the androgen(s) responsible remain ambiguous. Recent studies have suggested that 11‐oxygenated C_19_ steroids (11‐oxyandrogens), specifically 11‐ketotestosterone, may be a good marker for hyperandrogenism in PCOS.

**Objective:**

To investigate the utility of 11‐oxyandrogens to differentiate women with and without PCOS relative to classical androgens.

**Design Setting:**

Case–control study performed at a PCOS clinic and research center in an academic setting.

**Patients:**

114 women with PCOS and 78 healthy controls.

**Interventions:**

Using the PCOS Tissue Bank, serum samples and data from 114 women registered from 2013 to 2017 between the ages of 18–40 years, were obtained and classified using Rotterdam PCOS criteria. Data were compared to 78 healthy women of similar age, with serum samples obtained between 2017 and 2020. 11‐oxyandrogens and sex steroids were measured using mass spectrometry, and their associations to Rotterdam PCOS, age, and BMI were assessed.

**Main Outcome Measures:**

11‐oxyandrogens and sex steroids.

**Results:**

Total testosterone, androstenedione, and four 11‐oxyandrogens were significantly elevated in women with PCOS compared to age matched controls, controlling for age and BMI (*p* < 0.01 for all). When considered together, the four 11‐oxyandrogens were more predictive of PCOS compared to testosterone and androstenedione. When all androgens were considered individually, 11‐ketoandrostenedione was the most predictive of PCOS. Of the six androgens studied, 11‐ketotestosterone was the only androgen that demonstrated a weak association with hirsutism score (*r* = 0.17; *p* = 0.07) within the PCOS group.

**Conclusion:**

11‐oxyandrogens were statistically higher in women with PCOS and may serve as better predictors of PCOS than testosterone and androstenedione.

## Graphical abstract

Results from our research are unique from previous published data, in that we found 11‐ketoandrostenedione a far better predictor of polycystic ovary syndrome (PCOS) than classical androgens such as testosterone and androstenedione. Our findings also indicate that there may be population differences in concentrations of 11‐oxyandrogens in women with PCOS globally.

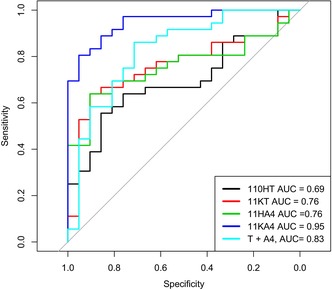

**Summary**

- 11‐Oxygenated C_19_ steroids are higher in women with polycystic ovary syndrome (PCOS) compared to age matched healthy controls and appear to be better predictors of PCOS than the classical androgen measurement of testosterone.

## 1 Introduction

Polycystic ovary syndrome (PCOS), a disorder consisting of hyperandrogenism, menstrual dysfunction and ovarian morphology changes, continues to be the most common endocrine condition in women of reproductive age [1]. With estimations of anywhere from 5% to 20% prevalence worldwide, understanding the hormonal dysregulation and origin of androgen excess in this disorder has been a growing field of research [2, 3, 4].

Currently there are three well‐established society guidelines for diagnosing PCOS: National Institute of Health (NIH)/National Institute of Child Health and Human Development (NICHD) 1990 criteria, European Society of Human Reproduction and Embryology (ESHRE)/American Society for Reproductive Medicine (ASRM) Rotterdam 2003 criteria, and Androgen Excess PCOS (AE‐PCOS) Society 2006 criteria [4]. The common feature among these three diagnostic guidelines is the presence of either clinical or biochemical hyperandrogenism. Biochemical hyperandrogenism has classically been defined as either a total testosterone or calculated free testosterone level above assay reference range. While serum testosterone levels remain the gold standard marker for hyperandrogenemia, this hormone can be difficult to measure accurately due to low circulating concentrations in women and radioimmunoassays with poor sensitivity and specificity. Assay advancements with liquid chromatography tandem mass spectrometry (LC–MS/MS) have not fully resolved the quandary of interpreting normal testosterone levels in women with classic clinical findings of hyperandrogenism on exam. Indeed, prior investigators have reported inconsistent correlations between standard measures of hyperandrogenism, such as testosterone, and clinical hyperandrogenism [5]. This inconsistency has motivated interest in identifying alternative sources of androgen activity beyond classic measures.

While the source of androgen production in PCOS has been controversial, the most accepted theory is that both the ovaries and the adrenals contribute to androgen excess. In regards to adrenal sources, an increased abundance of four 11‐oxygenated C_19_ steroids (11‐oxyandrogens) including 11‐ketotestosterone, 11‐ketoandrostenedione, 11β‐hydroxytestosterone, and 11β‐hydroxyandrostenedione have been noted in several disorders of androgen excess, including premature adrenarche and congenital adrenal hyperplasia, as well as in states of high lipodystrophy and insulin resistance [6, 7]. However, the role for these precursors in PCOS has only recently been explored [8, 9, 10]. Of particular interest is 11‐ketotestosterone, which has similar androgenic activity to that of testosterone in in vitro studies [8, 11] and can be converted peripherally via 5‐alpha reductase to 11‐ketodihydrotestosterone, a potent androgen with similar activity to dihydrotestosterone (Figure S1) [12, 13]. To date, one European study has investigated 11‐oxyandrogens in PCOS and reported increased circulating levels compared to controls [10]. While this study highlighted the potential for new androgen markers, few investigations have confirmed these findings, especially in other ethnic populations.

The study described herein sought to compare the levels of 11‐oxyandrogens and classical androgens such as testosterone and androstenedione in those with and without PCOS and to test the hypothesis that 11‐oxyandrogens could serve as a predictive tool for PCOS.

## 2 Materials and Methods

This was a case–control study of subjects with and without PCOS. Full ethical approval was obtained by the UCSF institutional review board. All participants provided written informed consent prior to serum and data collection.

### 2.1 Study Populations

*PCOS*: One hundred and twenty‐nine well‐characterised individuals seen between 2013 and 2017 at a Multidisciplinary PCOS clinic and enrolled in a cohort study were sequentially selected. From these, 114 subjects were identified who met PCOS by Rotterdam criteria (see below). Subjects were 18–39 years of age and had been referred to the clinic for evaluation and management of PCOS. Common concerns included menstrual irregularity, clinical findings of hyperandrogenism or biochemical hyperandrogenism. Women with nonclassical congenital adrenal hyperplasia (NCAH), prolactin excess and thyroid dysfunction were excluded. Initial hormonal evaluations for PCOS were done off any hormonal therapy for at least a period of 1–3 months. Systematically collected patient data in the PCOS cohort database, including modified Ferriman Gallwey score, self‐reported ethnicity, transvaginal ultrasound and commercially obtained laboratory values (including fasting metabolic labs, estrone and dehydroepiandrosterone‐sulfate (DHEA‐S)), were also utilised. *Controls*: Seventy‐eight healthy women controls, 18–40 years of age, with no findings of PCOS, documented systemic steroid use, menstrual cycle abnormalities, hypogonadism, or active malignancies with previously quantified 11‐oxyandrogens, testosterone and androstenedione values were identified. Samples were obtained from the University of Michigan between 2017 and 2020.

### 2.2 Serum Collection

Serum from PCOS patients was collected as per the tissue bank protocol. After an initial evaluation in the multidisciplinary PCOS clinic, consenting participants underwent a 12‐h fasting blood draw between 8 and 11 am. All subjects who were previously on hormonal contraception had discontinued their medication for a minimum of one month prior to this assessment. Blood was processed by allowing a minimum of 30 min to clot, followed by centrifugation at 3000 rpm for 10 min. Using a pipette, 1.0 mL aliquots were separated into individual cryovials and then stored in a freezer at −80°C until the time of hormone testing. Serum samples for healthy women were collected between 7 and 10 am and processed similarly to controls described by Davio et al. [14].

### 2.3 Rotterdam Criteria

PCOS was diagnosed using Rotterdam criteria, requiring women to have at least two out of three criteria met: (1) presence of oligomenorrhoea or anovulation, (2) evidence of hyperandrogenism, or (3) polycystic ovarian (PCO) morphology changes as noted on ultrasonography [15, 16]. Hyperandrogenism was further categorised by either the presence of elevated free or total testosterone above the lab reference range and/or hirsutism on exam. A modified Ferriman‐Gallwey score (mFG) ≥ 8 as determined by a single dermatologist at the time of diagnosis signified medically significant hirsutism. In addition to the above criteria, patients were classified as being oligomenorrheic or non‐ovulatory based on menses occurring < 21 days or > 35 days apart. For those women who had recently taken a hormonal contraceptive prior to their initial evaluation, oligomenorrhoea was determined based on self‐reported menstrual history before starting any hormonal therapy and blood work was obtained following a minimum of 1 month off of hormonal therapy Additional baseline measures and demographics were obtained using the tissue bank database.

### 2.4 Hormone Analysis

All steroid measurements (including testosterone, 11‐ketotestosterone, androstenedione, 11‐ketoandrostenedione, 11β‐hydroxyandrostenedione and 11β‐hydroxytestosterone) were performed using LC–MS/MS at the University of Michigan, as previously described [14, 17].

### 2.5 Statistical Analysis

Characteristics and hormone measures for patients with and without PCOS were compared using descriptive statistics, with *p*‐values that were computed from two‐sided t‐test using the mean equivalence between the two groups. Subsequently, hormone measures were compared using multivariate linear regression analyses controlling for age and BMI. To assess correlations between androgen levels and select PCOS characteristics, Pearson correlation coefficients were calculated. To determine the predictive capacity of androgen measures, we performed cross‐validation procedures and constructed receiver operating characteristic (ROC) curves. In particular, we first randomly selected 135 (70% of the sample) samples as the training data and saved the rest of the data as the testing data. The 70%–30% partition was selected to ensure that there are sufficient samples in the training data for the parameter estimation and sufficient case and control samples in the testing data to evaluate the prediction sensitivity and specificity. Using training data, we built a logistic regression model (model 1) between the group membership (PCOS vs. control) and predictors 11β‐hydroxytestosterone, 11‐ketotestosterone, 11β‐hydroxyandrostenedione, 11‐ketoandrostenedione, age and BMI. We then utilised the fitted model to predict the group membership in testing data. Similarly, we built a logistic regression model (model 2) with testosterone, androstenedione, with age and BMI as predictors, and performed the same training and testing procedures. We then constructed ROC curves based on both model prediction results, where the thresholds used to construct ROC curves were selected as the means between any two consecutive predicted linear scores from the logistic regression model [18]. We performed this cross‐validation for both models one hundred times and compare the area under the ROC curve in each iteration. Spearman correlations were used to study correlation between BMI and the adrenal steroids. All testing was performed at the two‐tailed 0.05 level of significance.

## 3 Results

Out of 129 patients sequentially selected from the PCOS tissue bank, 114 women met Rotterdam criteria and were included in our PCOS group. PCOS subjects had a median age of 27 years, with a range of 18–39 years. The majority (67%) had all three Rotterdam features (oligomenorrhea, hyperandrogenism and PCO morphology; phenotype A; *n* = 76); 3% had oligomenorrhea and hyperandrogenism (phenotype B; *n* = 4); 22% had hyperandrogenism and PCO morphology (phenotype C; *n* = 25) and 8% had oligomenorrhea and PCO morphology (phenotype D; *n* = 9). Of the 78 healthy controls, the median age was 31 years, with a range of 18–40 years. As expected, BMI was significantly higher among our PCOS cohort compared to controls (median 28.3 vs. 24.7 kg/m^2^, *p* = 0.026). Additional characteristics are shown in Table 1.

**TABLE 1:** Baseline characteristics of the PCOS cohort and healthy female controls.

	PCOS (*n* = 114)	Controls (*n* = 78)	*p*
Age (years)	27 (24, 32)	31 (25, 35.8)	0.0023
BMI (kg/m^2^)	28.3 (23.6, 34.3)	24.7 (22.2, 31.9)	0.026
Ethnicity (%)	Caucasian/European (47%) Asian (15%) African American (4%) Hispanic (10%) Middle Eastern (3%) Other or mixed ethnicity (16%) No reported ethnicity (5%)		
PCOS Phenotype (%) (*n* = 114)			
A = Oligomenorrhoea + Hyperandrogenism + Polycystic ovarian morphology (*n* = 76)	67%		
B = Oligomenorrhoea + Hyperandrogenism (*n* = 4)	3%		
C = Hyperandrogenism + Polycystic ovarian morphology (*n* = 25)	22%		
D = Oligomenorrhoea + Polycystic ovarian morphology (*n* = 9)	8%		
Modified Ferriman Gallwey score (*n* = 112)	10 (7, 14)		
Mean follicle count per ovary (*n* = 101)	26 (17, 38)		
Mean ovarian volume (mL) (*n* = 85)	8.1 (6.3, 11.2)		
Systolic blood pressure (mmHg) (*n* = 111)	119 (112, 127)		
Diastolic blood pressure (mmHg) (*n* = 111)	71 (65, 79)		
Waist Circumference (cm) (*n* = 107)	85.8 (73.7, 96.5)		
Haemoglobin A1c (%) (*n* = 73)	5.4 (5.2, 5.5)		
Testosterone (ng/dL)	44.6 (35.00, 59.60)	30.450 (22.90, 39.03)	< 0.0001
Androstenedione (ng/dL)	158.8 (120.65, 210.675)	87.45 (65.05, 114.18)	< 0.0001
11β‐hydroxytestosterone (ng/dL)	17.40 (12.90, 21.95)	11.35 (6.90, 16.30)	< 0.0001
11β‐hydroxyandrostenedione (ng/dL)	239.7 (155.5, 346.4)	117.45 (85.13, 151.60)	< 0.0001
11‐ketotestosterone (ng/dL)	35.8 (23.1, 48.2)	21.45 (16.20, 31.63)	< 0.0001
11‐ketoandrostenedione (ng/dL)	47.050 (26.425, 85.025)	14.700 (12.25, 20.425)	< 0.0001

*Note:* Values are median (interquartile) or percentages. *p*‐value is from Wilcoxon signed rank test.

### 3.1 Classical and 11‐Oxyandrogens in PCOS and Controls

All four 11‐oxyandrogens were significantly higher in the PCOS cohort compared to controls, as were testosterone and androstenedione (Table 1). The magnitude and significance of these differences were robust to adjustment for age and BMI (Table 2). In considering standardised regression coefficients, androstenedione and 11‐ketoandrostenedione demonstrated the strongest relationship with PCOS status, which we found to be statistically significant (Table 2).

**TABLE 2:** Estimated differences in serum androgens (SE) between PCOS and controls.

Serum androgen	Coefficient (ng/mL)	95% CI	Standardised coefficient	*p*
11β‐hydroxytestosterone	4.84 (1.30)	[2.305, 7.375]	0.27	0.0002
11‐ketotestosterone	13.93 (2.79)	[8.4895, 19.3705]	0.35	< 0.0001
11β‐hydroxyandrostenedione	130.87 (22.04)	[87.892, 173.848]	0.41	< 0.0001
11‐ketoandrostenedione	43.88 (5.31)	[33.5255, 54.2345]	0.43	< 0.0001
Testosterone	17.12 (3.02)	[11.231, 23.009]	0.40	< 0.0001
Androstenedione	88.63 (10.75)	[67.6675, 109.5925]	0.53	< 0.0001

*Note:* Differences adjusted for age and BMI.

### 3.2 11‐Oxyandrogens as Predictors of PCOS

**FIGURE 1:**
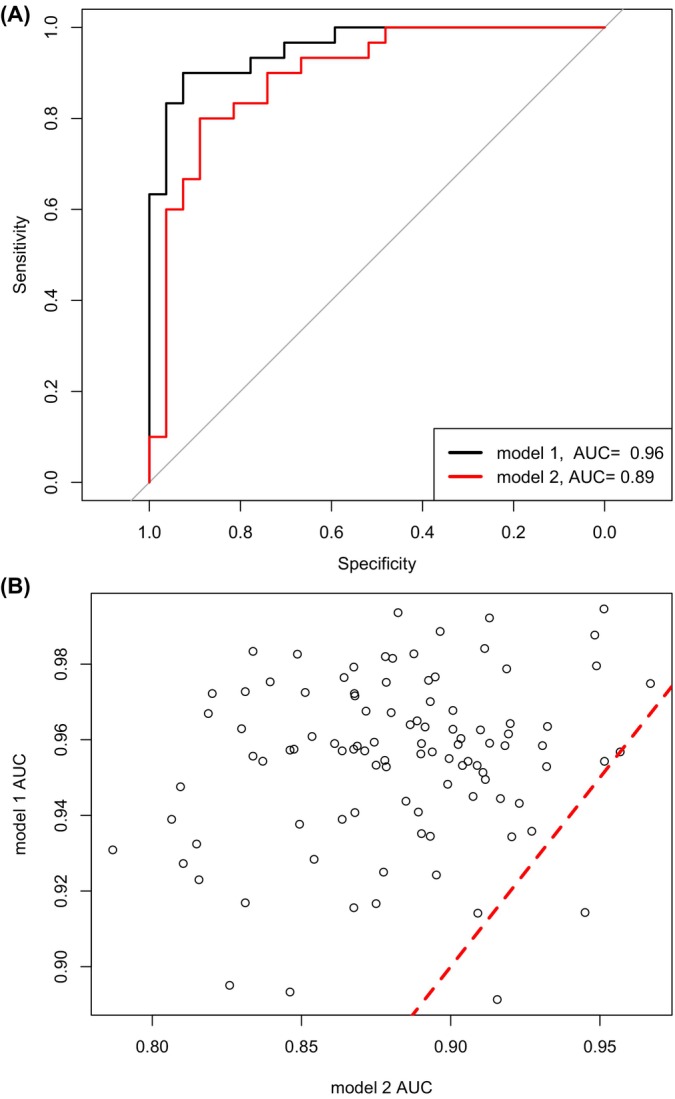
Prediction of PCOS for Models 1 and 2. **(A)** Receiver operator curves for Models 1 and 2 demonstrate that Model 1 has increased area under the curve (AUC) compared to Model 2. **(B)** Comparison of AUC in 98 out of 100 experiments, Model 1 yields an increased AUC compared to Model 2. The points left of the dash‐line represent the experiments where the Model 1 [2] yield better AUC. Model 1 includes 11‐hydroxytestosterone, 11‐ketotestosterone, 11‐hydroxyandrostenedione (11OHA4), 11‐ketoandrostenedione (11KA4), age and BMI. Model 2 includes Testosterone (T) and Androstenedione (A4), age and BMI. The data was resampled 100 times, resulting in a median AUC (95% CI) of 0.960 (0.914, 0.992) for Model 1 and 0.88 (0.860, 0.912) for Model 2.

**FIGURE 2:**
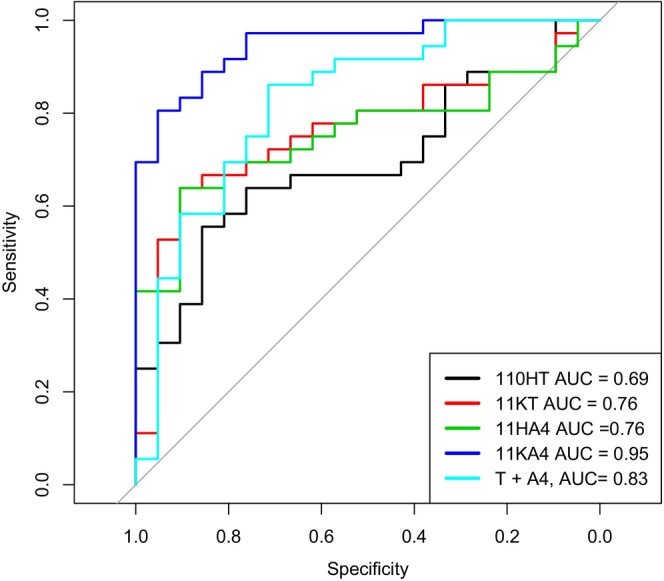
AUC comparisons of each androgen in predicting PCOS. ROC curves for each of the 11‐oxyandrogens and classical androgens from one resampling procedure. 11KA4 had the highest AUC among all the predictors. 11OHT, 11‐hydroxytestosterone; 11KT, 11‐ketotestosterone; 11OHA4, 11‐hydroxyandrostenedione; 11KA4, 11‐ketoandrostenedione; T, Testosterone; A4, androstenedione. Results over 100 resampling yields median 11HT AUC (95% CI) = 0.7064 (0.591, 0.816), median 11KTAUC = 0.754 (0.664, 0.852), median 11OHA4 AUC = 0.771 (0.683, 0.888), median 11KA4 AUC = 0.95 (0.880, 0.980) and median T + A4 AUC = 0.88 (0.860, 0.912).

We next sought to determine whether 11‐oxyandrogens are better predictors of PCOS compared to classical androgens. We first randomly selected 70% of subjects for a training cohort and 30% for a testing cohort. We constructed a logistic regression model between the group membership (PCOS or control) and 11β‐hydroxytestosterone, 11‐ketotestosterone, 11β‐hydroxyandrostenedione, 11‐ketoandrostenedione, age and BMI based on training cohort data (Model 1). A similar logistic regression model was constructed between group membership and testosterone, androstenedione, age and BMI (Model 2). We utilised the fitted models to predict the group membership using the testing data and constructed ROC curves. These analyses found that the 11‐oxyandrogens (Model 1) had better prediction accuracy compared to classical androgens (Model 2) (Figure 1A). After performing the cross‐validation procedure one hundred times and plotting the AUCs for both models, we found the median AUC (95% CI) of 0.96 (0.914, 0.992) for Model 1 and 0.88 (0.86, 0.912) for Model 2. Since the 95% CI for Model 2 does not include the median value of Model 1, Model 1 demonstrates a significantly better AUC than Model 2 (Figure 1B). Taken together, these results demonstrate that 11‐oxyandrogens as a group may predict PCOS better than classical androgens. Finally, using a similar strategy, we constructed ROC curves on each 11‐oxyandrogen to see which individually would best predict PCOS and found that 11‐ketoandrostenedione was the most predictive hormone (AUC = 0.95), out‐performing a combination of testosterone and androstenedione (AUC = 0.83) (Figure 2). Results over 100 resampling yields median 11HT AUC = 0.7064 (0.591, 0.816), median 11KT AUC = 0.754 (0.664, 0.852), median 11OHA4 AUC = 0.771 (0.683, 0.888), median 11KA4 AUC = 0.95 (0.880, 0.980) and median T + A4 AUC = 0.88 (0.860, 0.912).

### 3.3 11‐Oxyandrogens and PCOS Phenotypes

In an exploratory analysis, controlling for age and BMI, we found that 11‐oxyandrogens did not show significant differences across PCOS phenotypes (Table 3). In contrast, both testosterone and androstenedione were significantly lower in subjects with ovulatory PCOS (phenotype C; polycystic ovaries and hyperandrogenism).

**TABLE 3:** Estimated differences (Coefficient ng/mL) in serum androgens (SE) between PCOS Phenotype A (referent) and phenotypes B, C and D.

Serum androgen	Phenotype B	Phenotype C	Phenotype D
11β‐hydroxytestosterone	2.1 (12.2)	−0.59 (5.3)	−0.57 (7.2)
11‐ketotestosterone	10.2 (5.0)	−0.12 (0.98)	−2.3 (7.6)
11β‐hydroxyandrostenedione	91.8 (0.5)	33 (33.7)	−86.3 (0.17)
11‐ketoandrostenedione	3.9 (26.0)	5.3 (10.3)	−28.4 (15.5)
Testosterone	−7.2 (13.4)	−14.2 (5.2)**	−11.5 (7.8)
Androstenedione	−16.8 (49.5)	−41.5 (19.5)*	−48.7 (39.4)

*Note:* Phenotype A = oligomenorrhoea, hyperandrogenism and polycystic ovaries; Phenotype B = oligomenorrhoea and hyperandrogenism; Phenotype C = hyperandrogenism and polycystic ovaries; Phenotype D = polycystic ovaries and oligomenorrhoea; all differences adjusted for age and BMI.

* *p* = < 0.10.

** *p* = < 0.05.

### 3.4 11‐Oxyandrogens and PCOS Characteristics

Finally, we explored relationships between serum androgens and select PCOS characteristics within the PCOS cohort (Table 4). Of all six androgens, only 11‐ketotestosterone showed a weak association with the modified Ferriman–Gallwey score (*r* = 0.17; *p* = 0.07). The 11‐oxyandrogens as well as testosterone were not related to BMI, although androstenedione demonstrated an inverse relationship with this measure (*r* = −0.24; *p* < 0.05). We did not find that fasting glucose or insulin was related to androgen levels in this cohort; however, both 11β‐hydroxyandrostenedione and 11‐ketoandrostenedione demonstrated a negative association with C‐reactive protein. We also found testosterone and androstenedione, but not the 11‐oxyandrogens, showed highly significant, positive relationships to ovarian follicle number and volume.

**TABLE 4:** Pearson correlations coefficients (*r*) for the relationship between serum androgens and select PCOS characteristics within the PCOS group.

|	11β‐hydroxy testosterone	11‐keto testosterone	11β‐hydroxy androstenedione	11‐keto androstenedione	Testosterone	Androstenedione
Age (years)	0.05	−0.03	−0.11	−0.01	−0.02	−0.15
BMI (kg/m^2^)	−0.11	0.01	−0.05	0.03	−0.10	−0.24**
Modified Ferriman Gallwey score	0.09	0.17*	0.12	0.11	−0.002	−0.05
Estrone (pg/mL)	−0.17	0.02	0.01	0.54*****	0.35****	0.36****
DHEA‐S (μg/dL)	0.30*	0.31*	0.13	0.01	−0.06	0.11
LDL (mg/dL)	−0.04	−0.05	−0.18*	−0.14	−0.04	−0.11
HDL (mg/dL)	0.09	0.03	0.10	0.12	0.09	0.21**
CRP (mg/L)	0.13	0.01	−0.26**	−0.31**	−0.11	−0.09
TG (mg/dL)	−0.15	−0.12	−0.13	−0.13	0.10	−0.04
Fasting insulin (mIU/mL)	−0.1937	−0.05	−0.021	−0.10	−0.05	−0.18
Fasting Glucose (mg/dL)	−0.05	−0.02	0.02	0.02	−0.04	−0.04
Mean ovarian volume (mL)	−0.03	−0.00	−0.05	−0.05	0.42*****	0.36*****
Mean follicle per ovary	−0.09	−0.04	−0.04	0.003	0.48*****	0.46*****

Abbreviations: CRP, C‐reactive protein; DHEA‐S, dehydroepiandrosterone; mFG, modified Ferriman Gallwey score; HDL, high‐density lipoprotein; LDL, low‐density lipoprotein; TG, triglycerides.

* *p* = < 0.10.

** *p* = < 0.05.

**** *p* = < 0.0001.

***** *p* = < 0.00001.

## 4 Discussion

We sought to compare 11‐oxyandrogens in a group of U.S. women with and without PCOS and to test the hypothesis that 11‐oxyandrogens can help distinguish between individuals with and without this disorder. We found that all four of these steroids are significantly elevated in PCOS, suggesting adrenal androgens may serve as an important source of androgen excess in PCOS. We also found that these androgens, considered together, serve as superior markers for predicting PCOS compared to the classical androgens, testosterone, and androstenedione. Furthermore, in considering each androgen individually, we found that 11‐ketoandrostenedione was the most discriminatory biomarker for PCOS, out‐performing testosterone and androstenedione.

Our study adds to a growing body of work regarding the role of 11‐oxyandrogens in PCOS. O'Reilly et al. was the first to show that individuals with PCOS exhibit higher concentrations of 11‐oxyandrogens compared to healthy female controls [10]. In their PCOS cohort (*n* = 114), they found 11‐ketotestosterone concentrations to be threefold higher than testosterone; however, median testosterone in this cohort was only 20 ng/dL, substantially lower than in other published reports [4]. In contrast, Yoshida et al. assessed Japanese women with (*n* = 28) and without (*n* = 31) PCOS and found that only 11β‐hydroxytestosterone was significantly elevated in the PCOS cohort [19]; however, their reported median concentrations of the 11‐oxyandrogens were markedly lower in comparison to O'Reilly et al.'s report, likely due to assay differences.

Recently, investigators had also sought to correlate 11‐oxyandrogens with metabolic features in general populations. In a study of 217 women with ages ranging between 18 and 95, 11β‐hydroxytestosterone was found to directly correlate with BMI, whereas testosterone, 11‐ketoandrostenedione and androstenedione were inversely associated to BMI [14]. These findings have largely been explained by the higher reductive 17β‐hydroxysteroid dehydrogenase activity and expression of aldo‐keto reductase type 1C3 (AKR1C3) in obese individuals as well as in women with PCOS [20, 21]. Based on these findings, we suspect that 11‐oxyandrogens may be both relevant to the hyperandrogenic state and insulin resistance seen in women with PCOS. However, in our study, within our PCOS group, we found that 11‐oxyandrogens were not related to BMI, although androstenedione was. The difference in our results may be due to our focus on a PCOS population, in which 11‐oxyandrogens were more broadly elevated. O'Reilly also found significant correlations between metabolic markers with several of the 11‐oxyandrogens in a population of both controls and PCOS. In analyses conducted within our PCOS cohort, we did not find evidence that metabolic markers, such as fasting insulin or lipids, shared a linear relationship with adrenal androgens. It is possible that the associations observed by O'Reilly et al. were confounded by PCOS status, as this evaluation was conducted in a group composed of both PCOS and controls. Notably, our results echo studies in general populations in showing a lack of decline in 11‐oxyandrogens with aging, suggesting excess adrenal androgen production may continue with women with PCOS after the menopausal transition.

A novel element of our study is our comparison of 11‐oxyandrogens across PCOS phenotypes. Although our results lack sufficient power for definitive conclusions, we did not observe any significant differences in 11‐oxyandrogen levels across PCOS phenotypes. In contrast, both androstenedione and testosterone were significantly lower in those with ovulatory PCOS (phenotype C: polycystic ovaries and hyperandrogenism). This may suggest that in those with normal ovarian function, adrenal androgens may serve as an important source of androgen excess.

The strengths of our study are its utilisation of a well‐characterised group of women with PCOS and a control population. To our knowledge, this is the first study to test the predictive ability of 11‐oxyandrogens to identify PCOS and the first to examine 11‐oxyandrogens across PCOS phenotypes. Nevertheless, several limitations should be considered. For one, our PCOS and control subjects were recruited from two distinct locations and therefore may be subject to selection bias. However, this concern is tempered by the fact that all androgen testing was performed at the same location. There is also the possibility that some of our control patients were misclassified and may have had ovulatory PCOS. Although these subjects had no documented menstrual cycle irregularities, they did not undergo extensive evaluation for PCOS with a pelvic ultrasound. However, misclassification would bias results toward the null, and a study that utilises controls in which PCOS was specifically excluded might observe larger differences in androgen levels. Another limitation is that detailed information, such as ethnicity and smoking, were not available for our control population, limiting our ability to include these possible confounders in multivariate analyses. Additionally, due to limited resources and blood samples we were unable to include further labs tests such as sex hormone binding globulin, AMH and free testosterone levels in our analysis. Although this was not formally assessed in our study, all women within the PCOS cohort had these levels checked including gonadotropins and metabolic screening labs prior to their diagnosis in our PCOS clinic, as part of their routine evaluation. However, as these tests were done prior to the study, using varied assays and laboratory locations, we did not feel we could use this data as part of our analysis. Finally, while all subjects had been off oral contraceptives for at least one month, the exact amount of time off hormonal therapy was not available and thus could also not be considered in our analyses. In summary, further large‐scale studies are needed to properly evaluate the role of 11‐oxyandrogens with respect to PCOS, and we view this manuscript as a preliminary investigation into this topic. Such studies should interrogate how these hormones vary with ethnicity, lifestyle factors and metabolic outcomes. Additionally, future work to harmonise LC–MS/MS assays will be critical to advancing the potential for these markers to improve diagnostics and care for women with PCOS.

## 5 Conclusions

In summary, our study showed that all four 11‐oxyandrogens are significantly increased among US women with PCOS compared to healthy women and appear to be better predictors of PCOS than classical androgen measurements. Further large‐scale studies are needed to confirm if 11‐oxyandrogens, specifically 11‐ketoandrostenedione, represents a superior single measure of hyperandrogenism in women with PCOS compared to testosterone. Additionally, studies are needed to determine how 11‐oxyandrogens contribute to the reproductive and metabolic physiology of PCOS throughout the life course, particularly at puberty and after menopause.

## Author Contributions

**Armaiti Parvez Mody:** conceptualization (equal), data curation (equal), formal analysis (equal), investigation (equal), methodology (equal), project administration (equal), resources (equal), supervision (equal), visualization (equal), writing – original draft (equal), writing – review and editing (equal). **Maya Beth Lodish:** conceptualization (equal), data curation (equal), formal analysis (equal), funding acquisition (equal), investigation (equal), supervision (equal), validation (equal), writing – review and editing (equal). **Richard Joseph Auchus:** conceptualization (equal), formal analysis (equal), methodology (equal), resources (equal), supervision (equal), writing – review and editing (equal). **Adina F. Turcu:** data curation (equal), formal analysis (equal), investigation (equal), resources (equal), writing – review and editing (equal). **Fei Jiang:** data curation (equal), formal analysis (equal), writing – review and editing (equal). **Heather Gibson Huddleston:** conceptualization (equal), data curation (equal), formal analysis (equal), funding acquisition (equal), investigation (equal), methodology (equal), project administration (equal), resources (equal), writing – review and editing (equal).

## Conflicts of Interest

The authors declare no conflicts of interest.

## Supporting information


**Figure S1.** Steroid pathway for 11‐oxyandrogens.

## Data Availability

The data that support the findings of this study are available from the corresponding author upon reasonable request.
