# Supplementary material for: Exploring the Predictive Role of 11‐Oxyandrogens in Diagnosing Polycystic Ovary Syndrome
Source: Endocrinol Diabetes Metab. 2025 Jan 15;8(1):e70022. doi: 10.1002/edm2.70022 (PMC11735743; doi:10.1002/edm2.70022)
Supplement: Supplementary file 1 — Figure S1. Steroid pathway for 11‐oxyandrogens. [file EDM2-8-e70022-s001.docx]

**Supplementary Figure 1. Steroid pathway for 11-oxyandrogens**


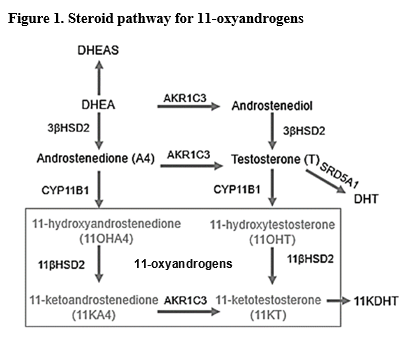


Abbreviations: Dehydroepiandrosterone sulfate (DHEAS), dehydroepiandrosterone (DHEA), Dihydrotestosterone (DHT), 11-ketodihydrotestosterone (11KDHT), 11-oxygenated C19 steroids (11-oxyandrogens)
